# Reliability of Canadian Emergency Department Triage and Acuity Scale (CTAS) in Saudi Arabia

**DOI:** 10.1186/s12245-015-0080-5

**Published:** 2015-08-07

**Authors:** Mustafa Alquraini, Emad Awad, Ra’ed Hijazi

**Affiliations:** Department of Anesthesia, Critical Care Medicine Program, Faculty of Health Sciences, McMaster University, 1280 Main St. W, Hamilton, ON L8S 4K1 Canada; Department of Emergency Medicine, King Abdulaziz Medical City for National Guard Health Affairs, P.O. Box 22490, Riyadh, 11426 Saudi Arabia; University of British Columbia, Okanagan, Community, Culture and Global Studies, Irving K. Barber School of Arts and Sciences, Art 270, 333 University Way, Kelowna, BC V1V 1V7 Canada

**Keywords:** Canadian triage and acuity scale (CTAS), Triage, Agreement, Reliability

## Abstract

**Background:**

The Canadian Emergency Department Triage and Acuity Scale (CTAS) is an integral part of the Canadian emergency medicine triaging system. There is growing interest and implementation of CTAS worldwide. However, little is known about its reliability outside Canada. The aim of this study was to determine the reliability agreement of CTAS in a tertiary care emergency center in Saudi Arabia.

**Methods:**

Ten triage nurses (five senior and five junior nurses) utilized CTAS guidelines to independently assign a triage level for 160 real case-based scenarios. Quadratic weighted kappa statistics were used to measure raters’ agreements.

**Results:**

Raters provided 1600 triage category assignments to case scenarios for analysis. Intra-rater agreement was similar for both senior and junior nurses; for senior nurses (SN1) kappa 0.871 95 % CI (0.840–0.897), and for junior nurses (SN2) kappa 0.871 95 % CI (0.839–0.898). Inter-rater agreement for the SN1 versus SN2 nurses had statistically meaningful agreement across different triage levels (weighted kappa = 0.770) 95 % CI (0.742–0.797).

**Conclusions:**

CTAS has good reliability among emergency department (ED) triage nurses in King Abdulaziz Medical City (KAMC), Saudi Arabia. The findings suggest that CTAS might be a reliable instrument when applied in countries outside Canada.

## Background

Triage systems aim to distribute medical resources according to patients’ needs [1]. Trained triage nurses are usually responsible for giving a triage level for patients’ conditions based on an established triage system within a short timeframe. Applying triage can lead to safe and efficient utilization of an emergency department (ED) [2]. The foundations of triage systems take into consideration the values of human life, health care resources, and fairness in distribution [3]. A valid and reliable triage instrument would serve these values. Most modern triage systems are reported to be valid; however, there are wide variations in their reliability (*k* = 0.25 to 0.91) [4]. Reliability of a triage system is of paramount importance toward achieving its stated goals. Different raters should come to the same decisions regarding patient prioritization to avoid unnecessary delay of treatment, especially as it has been shown that early treatment of certain conditions can improve outcomes [5].

There are several triage systems implemented around the world, such as the Canadian Emergency Department Triage and Acuity Scale (CTAS), the Emergency Severity Index (ESI), the Australian Triage Scale (ATS), and the Manchester Triage System (MTS) [4]. The CTAS triage system has been utilized in several leading health care institutes in Saudi Arabia for almost 10 years, including King Abdulaziz Medical City (KAMC), National Guard Health Affairs (NGHA). There are no national unified standards for triage system in Saudi Arabia. Currently, many Ministry of Health (MOH), non-MOH, and private Hospitals in Saudi Arabia are using CTAS [6].

Emergency physicians in Canada developed CTAS in the 1990s, and since then, it has been implemented in Canada and in other countries in the world [7]. CTAS is a five-level triage system (level I = resuscitation, level II = emergent, level III = urgent, level IV = less urgent, and level V = non-urgent) that is based on a list of patients’ presenting complaints with first- and second-order modifiers for specific conditions. Its principle operational objective is determining the time for the patient’s initial assessment by a physician. The implementation guidelines define a specific timeframe for a physician and a nurse assessment of patients according to the triage acuity scale (Table 1). The CTAS National Working Group (NWG) continues to revise the guideline, meeting annually and responding to feedback for quality improvements and standardization to achieve optimal reliability [8, 9]. In 2012, CTAS NWG determined that CTAS achieved steadiness and invariability [10]. However, concern was recently raised about the triage scoring reliability, after a study was conducted in Canada in which triage scoring agreements among nurses was disappointing: the inter-rater agreement of CTAS in that setting was moderate Kappa 0.44 (0.40–0.48) [11].

**Table 1:** CTAS time objectives

Triage level	Time for triage assignment	Time to nursing reassessment	Time to physician assessment
I	≤10 min	Continuous care	Immediate
II		Every 15 min	≤15 min
III		Every 30 min	≤30 min
IV		Every 60 min	≤60 min
V		Every 120 min	≤120 min

CTAS operational objectives can be achieved and implemented outside Canada [12]. Based on their study in Saudi Arabia, Elkum et al. found that CTAS could be implemented with achievable objectives, however the study was in a center with a special population, and it has not assessed CTAS reliability [13]. In this study, we aim to assess the inter-rater reliability of CTAS in our current clinical setting, which can serve as a base for benchmarking and future quality improvement in the triage process in KAMC as well as other hospitals in Saudi Arabia.

## Methods

The local institutional review board approved this study. We utilized electronically registered triage data for adult patients visiting the Emergency Care Center (ECC) at KAMC. KAMC is a tertiary care facility in Riyadh, Saudi Arabia. Its emergency care center is one of the largest in the Middle East with a capacity of 100 beds and more than 200,000 annual ED visits. Ten triage nurses (five senior and five junior nurses) were randomly selected by stratified randomization according to nurses rank and invited to participate in the study. Nursing ranking (senior vs. junior) in our institution depends on qualifications and developed countries experiences and not only to number of years of practice. All participants expected to have completed the mandatory triage course and be re-certified every 2 years. The triage course is based on CTAS 2008 guidelines and is delivered by our hospital. Case scenarios were extracted from real data of ED patient visits registered in QuadraMed’s Computerized Patient Record (QCPR) in the preceding 3 months before the start of the study. A total of 160 emergency patients’ assessment data was included (chief complaint, mode of arrival, vital signs, pain score, and general appearance), collected, and summarized as case scenarios on paper sheets. Patients’ visits were randomly selected through a two-stage stratified randomization by date and shifts in a retrospective pattern. Two investigators reviewed the case scenario answer sheets for data and language consistency and appropriateness. Pilot testing of answer sheets was done by a senior ED nurse who was not invited to participate in our study. After revision, we drafted the final version for the purpose of the study. Each rater independently assigned a triage level for 160 cases based on the information provided.

To measure raters’ agreement, we used Kappa statistics (*k*). For evaluating a scale with five categories (CTAS 1–5), the number of case scenarios was calculated by using a sample size calculator provided by Ineke van der Wulp [14], with К0 0.62, К1 0.70, *α* = 0.05, *β* = 0.80, and 10 raters. The strengths of agreement were determined by using Altman’s Agreement Criteria [15] (Table 2). All analyses were conducted using SAS version 9.2 (SAS Institute Inc., Cary, NC).

**Table 2:** Altman’s kappa strength interpretation criteria

*Ќ*	Strength of agreement
<0.20	Poor
0.21–0.40	Fair
0.41–0.60	Moderate
0.61–0.80	Good
0.81–1.00	Very good

## Results

All 10 participant nurses completed and submitted the data sheets, providing 1600 triage category assignments to case scenarios for analysis. Demographics and triage training background of the study cohort are summarized in Table 3.

**Table 3:** Demographics and training background of the study cohort

Variables	Senior nurses	Junior nurses	*p* value
	[n (%)]	[n (%)]	
Gender			
Male	3 (60)	0	0.166
Female	2 (40)	5 (100)	
Triage course taken			
Yes	5 (100)	5 (100)	
Last triage course taken			
<2 years	2 (40)	2 (40)	1.00
>2 years	3 (60)	3 (60)	
Number of years of practice [mean ± SE]	12.80 ± 1.93	11.40 ± 2.87	0.916

Raters triage agreement across all levels for cases was 52.31 % for resuscitation (CTAS I), 55.63 % emergent (CTAS II), 61.13 % urgent (CTAS III), 53.85 % less urgent (CTAS IV), and 56.14 % for non-urgent (CTAS V) level. Intra-rater agreement was similar for senior nurses SN1 kappa 0.871 95 % CI (0.84–0.897) and for junior nurses SN2 kappa 0.871 95 % CI (0.839–0.898). Inter-rater agreement for the senior (SN1) versus junior (SN2) nurses across different triage levels was weighted kappa 0.770 95 % CI (0.742–0.797) (Table 4).

**Table 4:** Kappa values for CTAS inter-observer agreement

Raters		95 % confidence interval (lower bound–upper bound)
Senior nurses (SN1) vs. junior nurses (SN2)	Simple kappa	
	0.632	0.591–0.673
	Weighted kappa	
	0.770	0.742–0.797

## Discussion

In our study, we assessed CTAS reliability by utilizing real case, paper-based scenarios rated by both senior and junior nurses in KAMC. Most of the cases triaged as urgent and less urgent (CTAS levels III and IV), which depict our ED census. However, our trial showed CTAS score distribution had highest agreement (61.13 %) for the urgent level (CTAS III). This finding is not congruent with previous studies that report the highest agreement across the extreme categories (e.g., CTAS level I) [16]. This could be explained by the fact that patients rated CTAS levels I and II, in our institution, are always assigned and shifted to the ED Critical Care (CC) area and seen almost always immediately. CTAS category V and most of level IV patients, on the other hand, are sent to the Urgent Care Center (UCC) in the ED. In addition, due to overcrowding, our nurses are not infrequently questioned by staff about their assignment, which places an implicit pressure on their decision. This ED design and patient flow may play a role in triage. Nonetheless, our results showed overall good reliability of the CTAS instrument.

For the second decade since its inception, CTAS is still being tested for its reliability. Most of the studies have been conducted in Canadian EDs with very few outside Canada. Our results suggest that CTAS has good reliability when applied in one of the major EDs in Saudi Arabia. Our local setting trial outcome is similar to previous Canadian studies that showed good reliability of CTAS [16, 17]. However, after CTAS’s major revision in 2004 followed by another in 2008 [8, 9], further studies showed less reliable results. Dallaire et al. found moderate agreement in their trial that compared base hospital nurses versus ED triage nurses [18], where base hospital nurses triaged the patients in the pre-hospital phase and then ED nurse re-triage them in ED. Their results could contradict our findings, perhaps due to time lag from pre-hospital to hospital arrival with the possibility of a change in patients’ condition during transport. In another study, Dallaire showed that CTAS inter-rater agreement was moderate when involving experienced nurses [11]. In our current trial, we had five senior and five junior nurses with considerable years of practice and experience, and more than half of them did not have recent triage course re-certification. Although there are differences in practice settings, this might open an avenue for further studies to assess the influence of rater’s experiences upon CTAS reliability. In addition, Fernandes found that applying the 2008 CTAS guideline yields moderate reliability compared to good reliability when using the former version of the triage guideline [19]. Our finding proved that CTAS has good reliability; however, we did not aim to compare previous CTAS guidelines to determine the effect on reliability.

### Limitations

In our study, we used paper-based case scenarios instead of real ED patient encounters. Although we copied the verbatim written visual clues in the triage notes to try matching the holistic patient assessment, real patient interaction may still influence triaging. Nonetheless, Worster et al. showed that using case scenarios is an acceptable alternative for triage reliability testing [20]. Another limitation for generalizability is conducting the study in one academic-affiliated ED. However, we believe that our setting represents an ideal ED in the region.

## Conclusions

In conclusion, CTAS has good reliability among ED nurses in KAMC, Saudi Arabia. The findings in our study suggest that CTAS might be a reliable instrument when applied in countries outside Canada. Further investigations may be warranted to explore the influence of rater’s experiences on the reliability of the updated CTAS guidelines.
